# Kaposi sarcoma of th e penis in anHIV-negative patient

**DOI:** 10.31744/einstein_journal/2019RC4504

**Published:** 2019-02-14

**Authors:** José Francisco Aguilar Guevara, Seila Lacarra Fernández, Oliver Rojas Claros, Pedro Giral Villalta, José Luis Cebrián Lostal, Miguel Angel Resano Abarzuza

**Affiliations:** 1 Complejo Hospitalario de Navarra, Pamplona, Navarra, Spain.; 2 Hospital Israelita Albert Einstein, São Paulo, SP, Brazil.

**Keywords:** Sarcoma, Kaposi, Penis/pathology, HIV, Penile neoplasms, Herpesvirus 8, human, Sarcoma de Kaposi, Pênis/patologia, HIV, Neoplasias penianas, Herpesvirus humano 8

## Abstract

Kaposi sarcoma is an angioproliferative disorder that ranges from a single indolent skin lesion to respiratory and gastrointestinal/visceral involvement. Kaposi sarcoma is rare in non-immunosuppressed patients. Nineteen cases of penile Kaposi sarcoma in HIV-negative patients were reported in 2012. We present the case report of a 48-year-old male patient with no previous medical history, who came to our urology clinic presenting a purple-color papule on the penis glans. Lab tests revealed negative serology for HIV, but tissue PCR was positive for human herpesvirus 8. Histopathology examination after lesion excision was compatible with Kaposi sarcoma. No other cutaneous or mucosal lesions were present. Primary Kaposi sarcoma of the penis is rare, but may occur in non-immunosuppressed patients.

## RESUMO

O sarcoma de Kaposi é uma doença angioproliferativa que varia de uma lesão cutânea indolente isolada ao envolvimento visceral respiratório e gastrintestinal. É raro em pacientes não imunossuprimidos. Dezenove casos de sarcoma de Kaposi de pênis em pacientes HIV negativos foram relatados em 2012. Descrevemos o caso de um paciente do sexo masculino, 48 anos, sem história pregressa, que se apresentou em nossa clínica urológica com pápula violeta na glande. Os testes de laboratório revelaram sorologia negativa para HIV, mas o PCR em tecido foi positivo para o herpesvírus humano 8. A histopatologia após a excisão da lesão foi compatível com sarcoma de Kaposi. Não existia outra lesão cutânea ou de mucosa. O sarcoma de Kaposi primário de pênis é raro, mas pode ocorrer em pacientes não imunossuprimidos.

## INTRODUCTION

Kaposi sarcoma (KS) is an angioproliferative disorder that requires human herpesvirus 8 (HHV-8) infection, also known as KS associated with herpesvirus (KSHV), for its development.^(^ ^1^ ^,^ ^2^ ^)^ There are four clinical-epidemiological forms of KS: classic (predominantly located in the lower extremities of elderly men from Mediterranean areas); endemic (in young Africans with frequent local invasive and/or visceral involvement); iatrogenic (associated with immunosuppressive drug therapy, typically reported in renal allograft recipients); and HIV-associated (epidemic).^(^ ^3^ ^)^

## CASE REPORT

**Figure 1:**
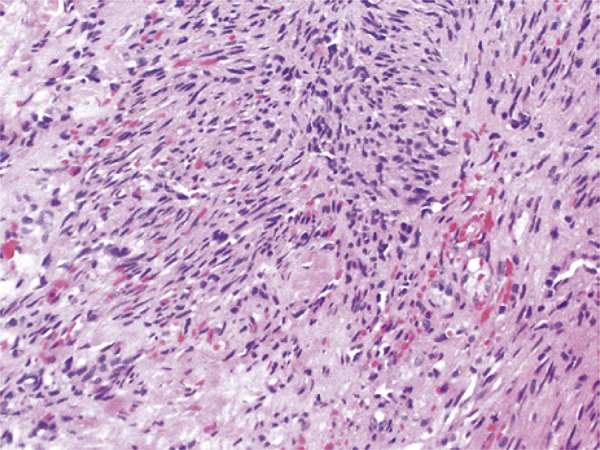
Spindle cell proliferation arranged in fascicles, forming vascular structures (hematoxylin and eosin, x20)

**Figure 2:**
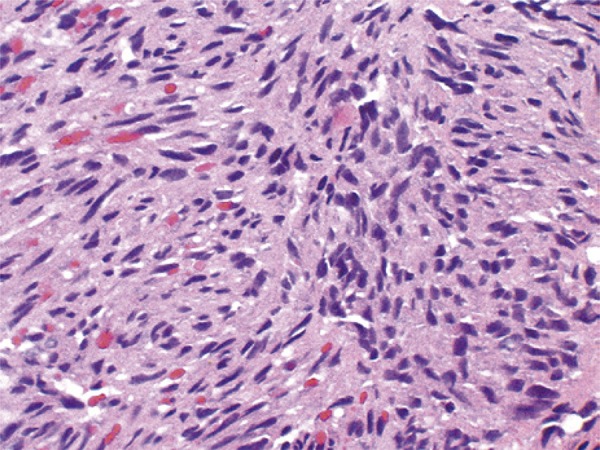
Spindle cells with minimal atypia and no mitosis

**Figure 3:**
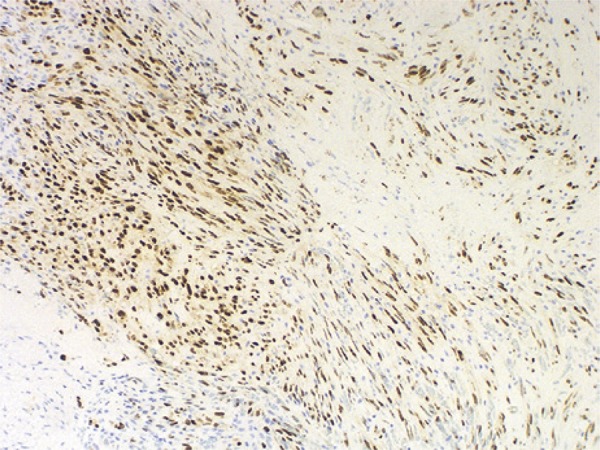
Positive human herpesvirus 8 nuclear staining

**Figure 4:**
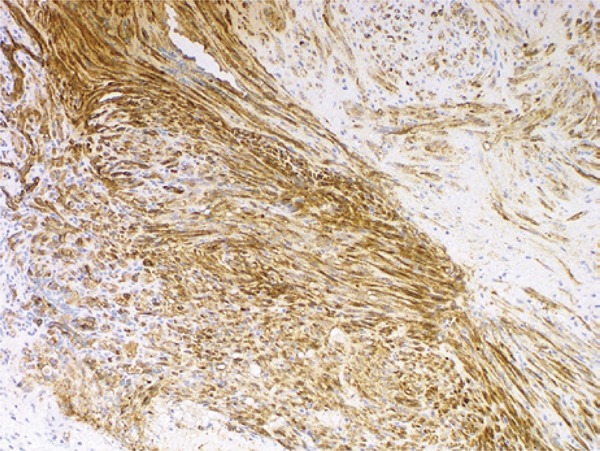
Expression of CD31

A 48-year-old male patient with no previous medical history presented to our urology clinic with a penile lesion of 1-month duration. The lesion was a purple-color papule over the glans near the urethral meatus, measuring approximately 1cm. There were no symptoms other than painful erection. No palpable inguinal or iliac lymph nodes were found. No other skin or mucosa lesions were observed during the physical examination. Laboratory tests included normal cell blood count, urine analysis and urine culture. Serologic testing for HIV, hepatitis B virus (HBV), hepatitis C virus (HCV) and treponemal tests were all negative. The lesion was excised, and the histologic examination revealed proliferation and fascicles of spindle cells associated with angiogenesis (Figures 1 and 2). Expression of CD31 and CD34 was detected, and positive HHV-8 nuclear staining was identified, all compatible with KS (Figures 3 and 4). Since the patient had not developed additional lesions, conservative treatment was chosen.

## DISCUSSION

Kaposi sarcoma is named after Moritz Kaposi, a Hungarian dermatologist and faculty member of the University of Vienna, who first described it in 1872 as an “idiopathic multiple pigmented sarcoma of the skin”.^(^ ^4^ ^)^ The disease ranges from a single indolent skin lesion to extensive respiratory and gastrointestinal visceral involvement. Kaposi sarcoma is classified in four types based on its clinical characteristics: (1) classic (originally described by Kaposi, which typically presents in middle-age or elderly patients), which has an indolent and rarely fatal course; (2) endemic (several forms described in Sub-Saharan Africans prior to the AIDS epidemic), which may present an indolent or aggressive disease course; (3) iatrogenic (associated with immunosuppressive drug therapy, typically seen in renal allograft recipients), which is often reversed by adjusting immunosuppressive agent doses; and (4) AIDS-associated (epidemic KS). Kaposi sarcoma is more prevalent in men and the reported male to female ratio is approximately 3:1. Few cases were described in individuals aged under 50 years.^(^ ^5^ ^)^ HHV-8 infection is required for the development of classic KS (CKS), but not all individuals infected develop the disease. In the Mediterranean area, *e.g.,* CKS develops annually in only 0.03% of HHV-8 infected men and only 0.01 to 0.02% of HHV-8 infected women aged over 50 years. This suggests the existence of cofactors that influence the risk for CKS after HHV-8 infection.^(^ ^6^ ^)^ Reddish-purple to bluish nodules are the primary presentation of penile KS. Other types of lesions, such as papules, plaques, and wart-like lesions are less common.^(^ ^7^ ^)^

Our patient presented primary penile KS by developing a purple papule in the glans, which seemed to be the classic type. Other lesions that may mimic the appearance of KS include bacillary angiomatosis, angiosarcoma, and benign vascular lesions, such as hemangiomas. Biopsy is required for the definitive diagnosis. In 2012, Fatahzadeh reported a total of only 19 cases with penile KS in HIV-negative patients.^(^ ^8^ ^)^ Three main pathologic stages have been described in the progression of KS lesions. In the patch stage, thin-walled vascular spaces are visible in the upper dermis with a sparse mononuclear cell infiltrate of lymphocytes, plasma cells, and macrophages. In the plaque stage, vascular spaces increase in number, the inflammatory infiltrate is more evident, and spindle cell bundles accumulate around the areas of angioproliferation. In the nodular stage, the tumor is more solid, and there are well-defined nodules, which consist of large fascicles of spindle-shaped endothelial cells with fewer and more compact vascular slits. The mononuclear cell infiltrate is no longer prominent, and few extravasated erythrocytes and macrophages are present between spindle cells. The lining cells of the clearly developed vascular structures are positive for vascular markers (such as factor VIII), while the spindle cells consistently stain for CD34 and commonly for CD31, but are negative for factor VIII.^(^ ^9^ ^)^ The main target of therapy is to decrease symptoms, reduce size and number of lesions and to delay disease progression. Many therapeutic approaches have been described, such as surgical excision, cryosurgery, radiation therapy, thermo-photoablation, laser therapy, local and systemic chemotherapy, and alpha and beta-interferon. Surgical excision is recommended for small and single lesions, whereas for multiple or large-size skin lesions radiation therapy can be recommended. Moreover, systemic chemotherapy has been employed in systemic forms.^(^ ^10^ ^)^ Conservative treatment was decided for our patient because he did not develop additional lesions on other sites. Treatment needs to be customized, based on a patient’s clinical and immunologic status.
